# Invasive Plant Suppresses the Growth of Native Tree Seedlings by Disrupting Belowground Mutualisms

**DOI:** 10.1371/journal.pbio.0040140

**Published:** 2006-04-25

**Authors:** Kristina A Stinson, Stuart A Campbell, Jeff R Powell, Benjamin E Wolfe, Ragan M Callaway, Giles C Thelen, Steven G Hallett, Daniel Prati, John N Klironomos

**Affiliations:** **1**Harvard Forest, Harvard University, Petersham, Massachusetts, United States of America; **2**Department of Integrative Biology, University of Guelph, Guelph, Ontario, Canada; **3**Division of Biological Sciences, University of Montana, Missoula, Montana, United States of America; **4**Department of Botany and Plant Pathology, Purdue University, West Lafayette, Indiana, United States of America; **5**Department of Community Ecology, UFZ Centre for Environmental Research, Halle, Germany; McGill UniversityCanada

## Abstract

The impact of exotic species on native organisms is widely acknowledged, but poorly understood. Very few studies have empirically investigated how invading plants may alter delicate ecological interactions among resident species in the invaded range. We present novel evidence that antifungal phytochemistry of the invasive plant, *Alliaria petiolata,* a European invader of North American forests, suppresses native plant growth by disrupting mutualistic associations between native canopy tree seedlings and belowground arbuscular mycorrhizal fungi. Our results elucidate an indirect mechanism by which invasive plants can impact native flora, and may help explain how this plant successfully invades relatively undisturbed forest habitat.

## Abstract 2

Experimental evidence reveals that an invasive species in North America--garlic mustard--negatively impacts the growth of native tree seedlings by disrupting native mycorrhizal mutualisms.

## Introduction

Widespread anthropogenic dispersal of exotic organisms has raised growing concern over their devastating ecological impacts, and has prompted decades of research on the ecology of invasive species [ 1– 3]. Exotic plants may become aggressive invaders outside their home ranges for a number of reasons, including release from native, specialized antagonists [ 4], higher relative performance in a new site [ 5], direct chemical (allelopathic) interference with native plant performance [ 6], and variability in the responses and resistance of native systems to invasion [ 7, 8]. Thus, successful invasion in many cases appears to involve the fact that invasive species are not at equilibrium, and are either freed of long-standing biotic interactions with their enemies in the home range, and/or disrupt interactions among the suite of native organisms they encounter in a new range [ 9]. Nevertheless, experimental data on species-level impacts of exotic plants are still limited [ 10]. One particularly understudied area is the potential for invasive plants to disrupt existing ecological associations within native communities [ 6, 10]. Many exotic and native plants alike depend upon mutualisms with native insects, birds, or mammals for pollination and seed dispersal [ 11], and with soil microbes for symbiotic nutrient exchange [ 12]. Thus, when an introduced species encounters a new suite of resident organisms, it is likely to alter closely interlinked ecological relationships, many of which have co-evolved within native systems [ 6, 11].

One such relationship is that between plants and mycorrhizal fungi [ 12]. Most vascular plants form mycorrhizal associations with arbuscular mycorrhizal fungi (AMF) [ 12], and many plants are highly dependent on this association for their growth and survival [ 12], particularly woody perennials and others found in late-successional communities [ 13]. In contrast, many weedy plants, in particular non-mycotrophic plants, can be negatively affected by AMF [ 14– 16]. Naturalized exotic plants have been found to be poorer hosts and depend less on native AMF than native plants [ 17]. They often colonize areas that have been disturbed [ 2], and disturbances to soil have been shown to negatively impact AMF functioning [ 18]. Furthermore, it has been proposed that the proliferation of plants with low mycorrhizal dependency may degrade AMF densities in the soil [ 17]. However, a few invasive plants proliferate in the understory of mature temperate forests [ 2], where AMF density is typically high [ 19]. The existing mycelial network in mature forest soils may facilitate the establishment of exotic, mycorrhizal-dependent, recruits [ 20, 21], but this should not be the case for non-mycorrhizal invaders. If non-mycorrhizal invasive plants establish and degrade AMF in mature forests, then the effects on certain resident native plants could be substantial.

One of the most problematic invaders of mesic temperate forests in North America is Alliaria petiolata (garlic mustard; Brassicaceae), a non-mycorrhizal, shade-tolerant, Eurasian biennial herb which, like most other mustards, primarily occupies disturbed areas. Garlic mustard is abundant in forest edges, semishaded floodplains, and other disturbed sites in its home range [ 22]. However, this species has recently become an aggressive and widespread invader of both disturbed areas and closed-canopy forest understory across much of the United States and Canada [ 23], where it apparently suppresses native understory plants, including the seedlings of dominant canopy trees [ 22, 24]. The mechanism underlying garlic mustard's unusual capacity to enter and proliferate within intact North American forest community has not yet been established.

As shown in recent greenhouse experiments, garlic mustard's impact on native understory flora may involve competitive [ 25] or allelopathic effects on native plants [ 26], but it has also been hypothesized that this species interferes with plant–AMF interactions in its invaded range [ 27]. Members of the Brassicaceae, including garlic mustard, produce various combinations of glucosinolate products [ 28], organic plant chemicals with known anti-herbivore, anti-pathogenic and allelopathic [ 29] properties, that may also prevent this non-mycorrhizal plant family from associating with AMF [ 30]. These phytochemicals may be released into soils as root exudates, as a result of damaged root tissue, or in the form of leaf litter. High densities of garlic mustard in the field correlate with low inoculum potential of AMF, and extracts of garlic mustard leaves have been shown to reduce the germination of AMF spores and impair AMF colonization of cultivated tomato roots in laboratory settings [ 27]. Although not all Brassicaceae are invasive, it is possible that garlic mustard's successful invasion of understory habitats involves the negative effects of its phytochemistry on the native plant and AMF species it encounters outside its home range. Others have shown that exotic plants can recruit different suites of microbial organisms in their new ranges that can be antagonistic to native plants [ 6]. However, to our knowledge, no previous studies have directly tested whether this species or any other exotic plant disrupts native plant–AMF mutualisms within natural communities. Here, we present novel evidence that garlic mustard negatively impacts the growth of AMF-dependent forest tree seedlings by its disruption of native mycorrhizal mutualisms. We further show that, because seedlings of dominant tree species in mature forest communities are more highly dependent on AMF than plants that typically dominate earlier successional communities, garlic mustard invasion may disproportionately damage mature forests relative to other habitats.

## Results/Discussion

We first tested whether native tree seedlings were less able to form mycorrhizal associations when grown in forest understory soils with a history of garlic mustard invasion than when grown in soils that had not experienced invasions (Experiment 1). We found that dominant native hardwood tree species of northeastern temperate forests, Acer saccharum (sugar maple), *Ac. rubrum* (red maple), and Faxinus americana (white ash), showed significantly less AMF colonization of roots ( Figure 1A) and slower growth ( Figure 1B) when grown in soil that had been invaded by garlic mustard. AMF colonization was almost undetectable in soil that had been invaded by garlic mustard. These reductions were similar to those observed when seedlings were grown in sterilized soil from both garlic mustard–invaded and garlic mustard–free sites ( Figure 1B), strongly suggesting that the mechanism by which garlic mustard suppresses the growth of native tree species is microbially-mediated, and not the result of soil differences or direct allelopathy.

**Figure 1:**
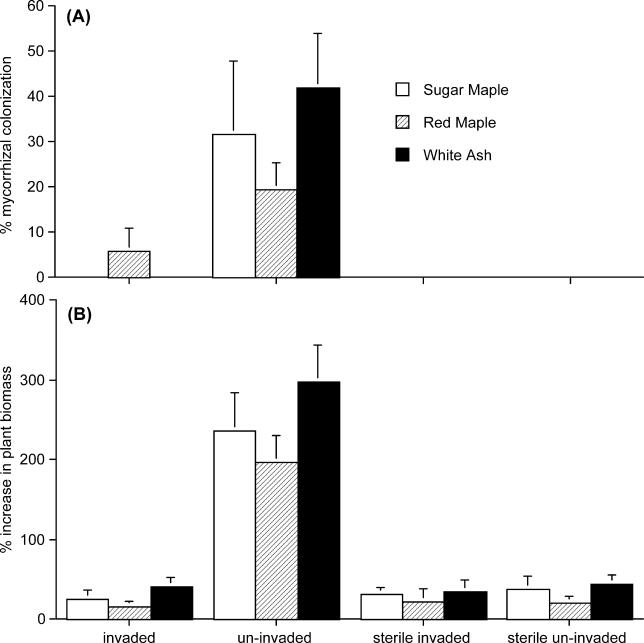
Experiment 1 The influence of field soils that were invaded or uninvaded by *Al. petiolata* (± sterilized) on (A) mycorrhizal colonization ( *F*_sugar maple_= 77.7, *df* = 3,39, *p* < 0.001; *F*_red maple_ = 60.5, *df* = 3,39, *p* < 0.001; and *F*_white ash_ = 116.6, *df* = 3,39, *p* < 0.001) and (B) biomass accumulation ( *F*_sugar maple_ = 57.8, *df* = 3,39, *p* < 0.001; *F*_red maple_ = 61.4, *df* = 3,39, *p* < 0.001; and *F*_white ash_ = 70.1, *df* = 3,39, *p* < 0.001) of native tree seedlings. Bars represent the mean and standard error.

We then conducted additional experiments to confirm that garlic mustard specifically caused AMF decline in the native soils (Experiment 2–4). We grew seedlings of the same three native tree species used in Experiment 1 in uninvaded forest soils that were conditioned for 3 mo with either garlic mustard plants or with one of the three native tree species. All three tree species demonstrated significantly lower AMF colonization in soils conditioned by *Al. petiolata* (0%–10%) than in soils conditioned by the native plants (20%–65%; Figure 2A). AMF colonization was similar in unconditioned (control) soils and soils conditioned with native plants. In addition, growth of the tree seedlings was the lowest in soils conditioned by garlic mustard ( Figure 2B), confirming that garlic mustard plants reduce native plant performance by interfering with the formation of mycorrhizal associations.

**Figure 2:**
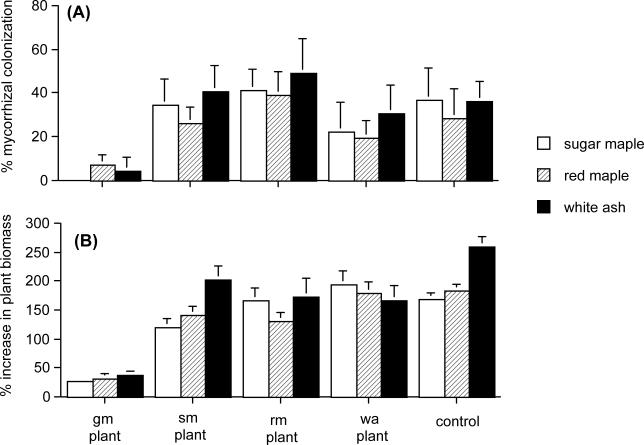
Experiment 2 The effect of soils conditioned with garlic mustard *Al. petiolata* (gm), sugar maple (sm), red maple (rm), or white ash (wa) on (A) mycorrhizal colonization ( *F*_sugar maple_ = 31.2, *df* = 4,49, *p* < 0.001; *F*_red maple_ = 18.2, *df* = 4,49, *p* < 0.001; and *F*_white ash_ = 22.1, *df* = 4,49, *p* < 0.001) and (B) increase in biomass ( *F*_sugar maple_ = 15.1, *df* = 4,49, *p* < 0.001; *F*_red maple_ = 18.1, *df* = 4,49, *p* < 0.001; and *F*_white ash_ = 13.2, *df* = 4,49, *p* < 0.001) of native tree seedlings. Bars represent the mean and standard error.

We investigated whether there is a phytochemical basis to garlic mustard's observed antifungal effects on AMF in Experiments 3–4. In an earlier study, Vaughn and Berhow [ 31] isolated the phytotoxic glucosinolate hydrolysis products allyl isothiocyanate, benzyl isothiocyanate, and glucotropaeolin from extracts of *Al. petiolata* root tissues and found evidence for their allelopathic effects on certain plants in the absence of mycorrhizas. These phytochemicals could have direct effects on plant growth through allelopathy as well as indirect effects via disruption of AMF. To experimentally establish that garlic mustard's effect on AMF is phytochemically based, we grew native tree seedlings on uninvaded soils to which we added individual aqueous extracts of garlic mustard or each of the native trees species (Experiment 3). We found that garlic mustard extract was just as effective as the living plant at reducing AMF colonization ( Figure 3A) and growth ( Figure 3B) of the native plants. Moreover, exposing AMF spores to extract of garlic mustard severely and significantly reduced germination rates of those spores (Experiment 4; Figure 3C). Collectively, our results clearly demonstrate that garlic mustard, probably through phytochemical inhibition, disrupts the formation of mycorrhizal associations. Our results thus reveal a powerful, indirect mechanism by which an invasive species can suppress the growth of native flora.

**Figure 3:**
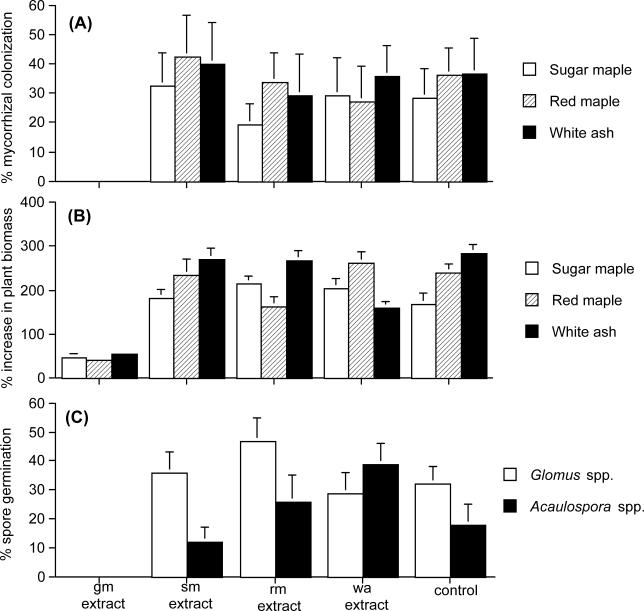
Experiments 3 and 4 The effects of extract of garlic mustard (gm), sugar maple (sm), red maple (rm), white ash (wa), or a water control on (A) mycorrhizal colonization of native tree seedlings ( *F*_sugar maple_ = 20.3, *df* = 4,49, *p* < 0.001; *F*_red maple_ = 19.8, *df* = 4,49, *p* < 0.001; and *F*_white ash_ = 25.4, *df* = 4,49, *p* < 0.001 [Experiment 3]), (B) increase in biomass of native tree seedlings ( *F*_sugar maple_ = 11.7, *df* = 4,49, *p* < 0.001; *F*_red maple_ = 14.2, *df* = 4,49, *p* < 0.001; and *F*_white ash_ = 27.9, *df* = 4,49, *p* < 0.001 [Experiment 3]), and (C) percent germination of native AMF spores ( *F _Glomus_* = 17.3, *df* = 4,49, *p* < 0.001; and *FA _caulospora_* = 21.8, *df* = 4,49, *p* < 0.001 [Experiment 4]). Bars represent the mean and standard error.

Because plants vary in their dependency on AMF [ 32], garlic mustard's disruption of native plant–fungal mutualisms should not inhibit the growth of all plants equally, but rather should correlate strongly with the mycorrhizal dependence of species encountered in the invaded range. Specifically, courser root production, which impedes the nutrient uptake of typically slow-growing, woody plants such as tree seedlings, may explain the stronger AMF dependency of certain species [ 19, 33]. To test whether garlic mustard's effects correlate with AMF dependency, and whether garlic mustard has stronger negative effects on forest tree seedlings than on other plants, we conducted another experiment (Experiment 5) using 16 plant species for which we determined AMF-dependency by computing the difference in plant growth in the presence and absence of AMF. We then tested the impact of garlic mustard on the AM fungal colonization and growth of each plant species as above. All 16 plants were successfully colonized by AMF, and the presence of garlic mustard heavily reduced AMF colonization in all plants ( Figure 4A). However, the presence of garlic mustard had a much stronger effect on plants that had high mycorrhizal dependency than those with less dependency ( Figure 4B). The strongest effects were observed for woody species most typically found in forested sites. These results indicate that the invasion of garlic mustard is more likely to negatively impact highly mycorrhizal-dependent tree seedlings than less-mycorrhizal-dependent plants. Thus, garlic mustard's successful colonization of understory habitat may be attributed in part to its ability to indirectly suppress woody competitors, and its effect on the native flora may be more detrimental in intact forests than disturbed sites. In addition, the data suggest that invasion by garlic mustard may have profound effects on the composition of mature forest communities (e.g., by repressing the regeneration of dominant canopy trees, and by favoring plants with low mycorrhizal dependency such as weedy herbs).

**Figure 4:**
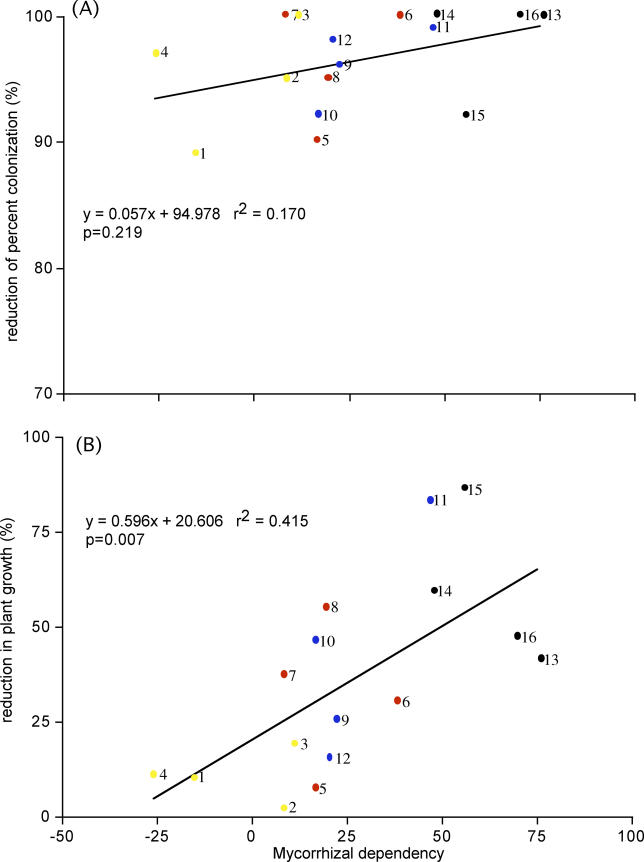
Experiment 5 (A) Effect of mycorrhizal dependency on *Al. petiolata* reduction of AMF colonization. (B) Effect of mycorrhizal dependency on *Al. petiolata* reduction in plant growth. Mycorrhizal dependency was calculated separately as the difference between plant growth in the presence and absence of AMF. Different colors represent plants with different life-history strategies, as follows: yellow dot, herbaceous colonizers of disturbed edges and bare ground; reddish brown dot, herbaceous edge and gap species; blue dot, woody colonizers of forest edges and gaps; black dot, tree species of mature forest. Species are labeled as follows (with mean mycorrhizal colonization in soil not conditioned by garlic mustard ± standard error in parentheses): 1 = *Ci. intybus* (18.5 ± 4.1), 2 = *Tr. repens* (46.7 ± 6.3), 3 = *Pl. major* (28.2 ± 3.7), 4 = *Ta. officinale* (37.3 ± 2.5), 5 = *S. canadensis* (48.0 ± 6.2), 6 = *C. leucanthemum* (34.6 ± 3.1), 7 = *D. carota* (40.4 ± 6.2), 8 = *As. syriaca* (52.1 ± 5.8), 9 = *J. virginiana* (31.2 ± 4.4), 10 = *Po. deltoids* (63.9 ± 4.5), 11 = *M. alba* (38.6 ± 5.9), 12 = *Pr. virginiana* (28.4 ± 4.2), 13 = *Fr. americana* (65.9 ± 5.3), 14 = *Ac. saccharum* (46.3 ± 3.7), 15 = *Ac. rubrum* (59.5 ± 5.7), 16 = *Pr. serotina* (34.8 ± 5.5).

In conclusion, our results reveal a novel mechanism by which an invasive plant can disrupt native communities: by virtually eliminating the activity of native AMF from the soil and drastically impairing the growth of native canopy species. It is currently unclear precisely which phytochemicals produced by garlic mustard have the observed antifungal properties, whether and how they interact with other soil microbes, and whether these anti-fungal effects extend to other functionally important forest soil fungi such as ectomycorrhizal fungi and saprotrophic fungi. In addition, within the home range, it is not known if evolutionary natural resistance of co-occurring European neighbors may buffer the effects of garlic mustard's antifungal properties [ 34– 36]. Further research in these directions is needed to better understand the effects of this invader on natural ecosystems and the mechanisms involved. In North America; however, the disruption of native tree seedling–AMF mutualisms may facilitate garlic mustard's invasion into mature forest understory and have particularly negative effects on the growth, survival, and recruitment of native trees, and the composition of forest communities.

## Materials and Methods

### Experiment 1

Using a 15-cm–wide corer, we collected soil from garlic mustard–invaded and nearby garlic mustard–free locations at each of five forested areas dominated by Acer rubrum L. (red maple), *Ac. saccharum* Marsh. (sugar maple), Fraxinus americana L. (white ash), and Fagus grandifolia Ehrh. (American beech) near Waterloo, Ontario, Canada. Invaded and uninvaded sites were randomly chosen within a 40-m ^2^ plot within each forested area. Soils from the invaded and uninvaded areas were pooled separately in the lab and screened to remove coarse roots and debris. Half the soil from each pool was then sterilized by autoclaving at 120 °C to create four soil treatments: (1) soil with a history of garlic mustard, (2) sterile soil with a history of garlic mustard, (3) soil without a history of garlic mustard, and (4) sterile soil without a history of garlic mustard. Six-inch pots were filled with a 1:1 mixture of sterilized silica sand and one of the four soil types. To each pot, we added a single seedling (seeds germinated on Turface [Aimcor, Buffalo Grove, Illinois, United States], a clay substrate) of one of the three native overstory tree species (sugar maple, red maple, or white ash) in a complete 4 × 3 factorial design with ten replicates of each treatment combination. The initial wet biomass of each seedling was recorded prior to planting, and dry weights were estimated using a dry–wet regression calculated from twenty extra seedlings. Pots were randomly placed on a greenhouse bench. Plants were watered (400 ml) once per week. Fertilizer was not added. After 4 mo of growth, shoots and roots were harvested, dried at 60 °C for 48 h, and weighed to determine biomass. An approximately 1-g subsample of roots from each seedling was extracted, stained with Chlorazol Black E [ 37] and analyzed for percent colonization by AMF [ 38]. Biomass and percent colonization data were analyzed using analysis of variance (ANOVA) for two fixed effects (soil type and species) and their interaction, followed by the Ryan-Einot-Gabriel-Welsch (REGW) multiple-range test.

### Experiment 2

Using field soil without a history of garlic mustard invasion (see Experiment 1), we grew garlic mustard, sugar maple, red maple, and white ash seedlings in separate 6-in pots ( *n* = 10) to condition the soil to each plant species. After 3 mo of conditioning, shoots and roots were removed. Unconditioned soil served as a control to the four plant-conditioning treatments. We added a single seedling of each of the three tree species to each of the five soil treatments. Pots were randomly placed on a greenhouse bench. Plants were watered (400 ml) once per week, without fertilizer. After 4 mo of growth, plants were harvested, biomass was determined, and percent mycorrhizal colonization of roots was assessed as in Experiment 1. Data were analyzed using ANOVA for two fixed effects (species and soil condition treatment). Means from the three species were pooled, and the effect of conditioning treatment was tested with a single-factor ANOVA followed by the REGW multiple-range test.

### Experiment 3

To 6-in pots containing field soil without a history of garlic mustard (see Experiment 1), we added a one-time, 100-ml aqueous extract [ 27] of whole plants of either garlic mustard, sugar maple, red maple, or white ash. A water control was included to give five treatments. Whole-plant extract was used to account for secondary compounds exuded through roots and leaf litter. After 1 wk of exposure to the extract, seedlings of each tree species were planted in each of these five treatments to give a full factorial design (extract source × tree species) with ten replicates of each treatment combination. Plants were watered (40 ml) every week, without fertilizer. After 4 mo of growth, plants were harvested, biomass was determined, and roots were assayed for mycorrhizal colonization as in Experiment 1. Data were analyzed by two-factor ANOVA.

### Experiment 4

Spores from AMF native to the forest sites were obtained using trap cultures (as described in [ 39], but with a mix of native plants) of soil samples from the uninvaded locations. We visually collected and separated *Glomus* and *Acaulospora* spores from these cultures, and compared germination rates of each genus in five treatments: a water agar control and water agar amended with an aqueous extract from each of the four plants, as above. Ten randomly drawn spores were added into each plate, which was then incubated at 18 °C for 10 d. Ten replicate plates were prepared for each of the ten treatment combinations (two AMF genera × five extracts). Plates were monitored microscopically for spore germination. Percent germination data were analyzed using ANOVA for two fixed effects (extract source and AMF genus), and because of a significant interaction, each AMF genus was then analyzed separately using single-factor ANOVA followed by the REGW multiple-range test.

### Experiment 5

We investigated the effects of garlic mustard on woody and herbaceous plants using the following 16 native plant species: *Cichorium intybus, Trifolium repens, Plantago major,* and Taraxacum officinale (dominant herbaceous colonizers of disturbed edges and bare ground); *Solidago canadensis, Chrysanthemum leucanthemum, Daucus carota,* and Asclepias syriaca (dominant herbaceous edge and gap species); *Juniperus virginiana, Populus deltoides, Morus alba,* and Prunus virginiana (dominant woody colonizers of forest edges and gaps); and *Fr. americana, Ac. saccharum, Ac. rubrum,* and *Pr. serotina* (dominant tree species of mature forest). Seedlings of each plant were transplanted into 8-in pots. For each species, growth was compared under the following soil treatments: (1) soil without a history of garlic mustard and inoculated with AMF, (2) soil without a history of garlic mustard, without AMF, and (3) soil with a history of garlic mustard, and inoculated with AMF. Experimental soil was collected within a mature-canopy maple forest from locations with and without garlic mustard. Soils from each location type were then mixed, cleaned of all coarse roots and debris, autoclaved, and added to the pots as a 1:1 mix of soil and silica sand. AMF spores were extracted from field soil collected from sites representing the four different habitats, and pooled. The AMF-inoculation treatment consisted of adding 200 randomly picked spores to each pot, 2 cm below the surface, and beneath the newly transplanted seedlings. Plants were watered (500 ml) once per week, without fertilizer. They were harvested after 4 mo of growth, dried at 60 °C for 36 h, and weighed to determine biomass. AMF dependency of each plant species was determined by computing the difference in plant growth in the presence and absence of AMF, i.e., contrast of treatments (1) and (2) [ 32]. The effects of garlic mustard on plant growth and percent colonization of each plant were determined by contrasting treatments (1) and (3). To ask whether any relationships existed among mycorrhizal dependency, life form, and garlic mustard effects, we performed two regressions: percent reduction in AMF colonization by garlic mustard on AMF dependency and percent reduction in plant biomass by garlic mustard on AMF dependency.

## Abbreviations

AMF: arbuscular mycorrhizal fungi
ANOVA: analysis of variance
REGW: Ryan-Einot-Gabriel-Welsch
