# Correction: Invasive Plant Suppresses the Growth of Native Tree Seedlings by Disrupting Belowground Mutualisms

**DOI:** 10.1371/journal.pbio.1001817

**Published:** 2014-02-27

**Authors:** The PLOS Biology Staff

In the Materials and Methods section, the amount of water used on the plants in Experiment 3 is listed incorrectly. The correct amount is: 400 ml.

## References

[1] Stinson KA, Campbell SA, Powell JR, Wolfe BE, Callaway RM, et al (2006) Invasive Plant Suppresses the Growth of Native Tree Seedlings by Disrupting Belowground Mutualisms. PLoS Biol 4(5): e140 doi:10.1371/journal.pbio.0040140 16623597 10.1371/journal.pbio.0040140 PMC1440938

